# Thoracic Stent Graft Implantation for Aortic Coarctation with Patent Ductus Arteriosus via Retroperitoneal Iliac Approach in the Presence of Small Sized Femoral Artery

**DOI:** 10.1155/2016/7941051

**Published:** 2016-05-03

**Authors:** Ozge Korkmaz, Osman Beton, Sabahattin Goksel, Hakkı Kaya, Ocal Berkan

**Affiliations:** ^1^Department of Cardiovascular Surgery, Cumhuriyet University, 58140 Sivas, Turkey; ^2^Department of Cardiology, Heart Center, Cumhuriyet University Hospital, Faculty of Medicine, Cumhuriyet University, 58140 Sivas, Turkey

## Abstract

Endovascular stent graft implantation is a favorable method for complex aortic coarctation accompanied by patent ductus arteriosus. Herein, an 18-year-old woman with complex aortic coarctation and patent ductus arteriosus was successfully treated by endovascular thoracic stent graft via retroperitoneal approach. The reason for retroperitoneal iliac approach was small sized common femoral arteries which were not suitable for stent graft passage. This case is the first aortic coarctation plus patent ductus arteriosus case described in the literature which is treated by endovascular thoracic stent graft via retroperitoneal approach.

## 1. Introduction

Coarctation of the aorta (CoA) is a common congenital heart disease that accounts for 5–8% of all congenital heart defects, and it occurs as a result of congenital narrowing of the aorta, which is typically located at the insertion of the ductus arteriosus just distal to the left subclavian artery [1]. Many cases may have a loss of luminal continuity between the ascending and descending aortic segments. Previous studies reported that the progression of the luminal stenosis may cause total occlusion of CoA [2, 3]. CoA could be accompanied by patent ductus arteriosus (PDA), bicuspid aortic valve, and ventricular septal defect. Endovascular stenting is a commonly preferred intervention for CoA [4–6]. Due to the coarcted segment in the aorta, blood flow distal to the coarcted segment decreases [7]. Common femoral artery diameter increases with age and body size, and females have smaller diameter of common femoral artery as compared to males [8]. Patients with small sized common femoral artery are not suitable candidates for endovascular interventions with big sized endovascular stent grafts. This potential difficulty may contribute to trying alternative ways such as retroperitoneal (RP) or transperitoneal (TP) approach for endovascular stent grafting. The RP approach was demonstrated to be associated with lower rates of postoperative complications when compared to the TP approach [9]. Herein, we presented a case of CoA associated with PDA who was successfully treated with thoracic endovascular stent graft via retroperitoneal iliac approach.

## 2. Case Report

**Figure 1:**
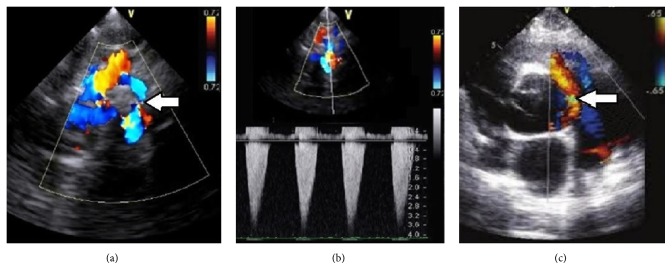
(a) Color flow Doppler imaging echocardiogram on the suprasternal longitudinal view showing coarcted segment of aorta with turbulence (arrow) in the descending aorta. (b) Continuous wave Doppler flow echocardiography demonstrating the coarctation gradient with a peak flow velocity in the descending aorta of 3.8 m/s which corresponds to a maximal gradient of 58 mmHg. (c) The two-dimensional echocardiogram, with color flow Doppler, recorded from the parasternal short-axis view, shows blood flow through a patent ductus arteriosus (arrow).

**Figure 2:**
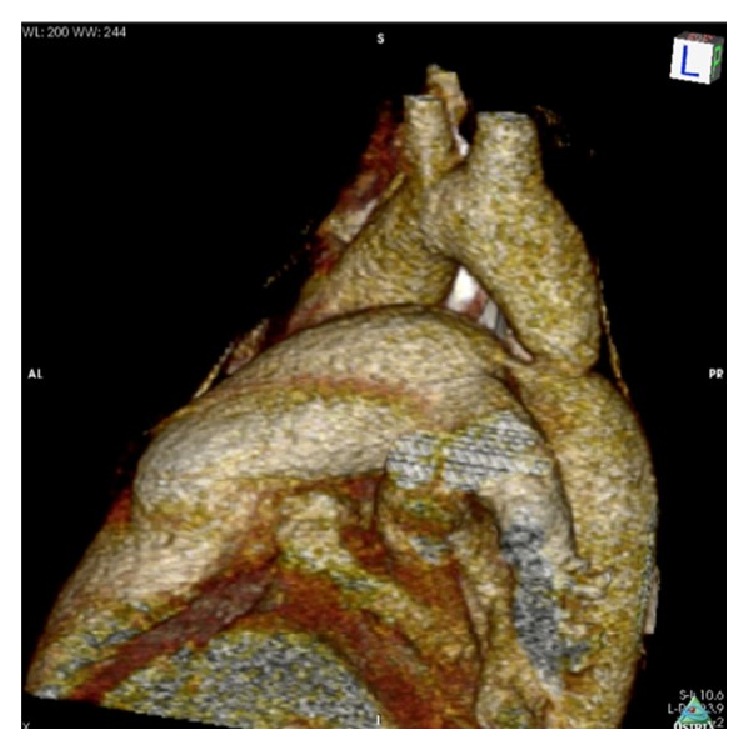
Thorax CT angiography, a strict aortic coarctation proximal part of descending aorta, and a small size patent ductus arteriosus were detected.

An 18-year-old woman was admitted to our hospital with breathlessness and hypertension. Her medical history included arterial hypertension for one year with no history of traumatic injury or Takayasu's arteritis. On physical examination, height was 150 cm and weight was 40 kg, pulse rate was 89 bpm with blood pressure of 162/95 mmHg in both arms, and all pulses were palpable. However, lower extremity pulses were diminished with a typical radial-to-femoral delay and systolic pressure difference between brachial and ankle was 80 mmHg. Routine blood tests and urine analysis were normal. A grade 3/6 systolic ejection murmur could be heard along the left intercostal area and severe S1 sound was audible. The examination of the respiratory and other systems was normal. Transthoracic echocardiography examination showed increased wall thickness of left ventricle (interventricular septum 1.3 cm and posterior wall 1.3 cm), normal left ventricular ejection fraction (65%), and increased pulmonary artery systolic pressure (45 mmHg). Aortic valve was bicuspid and normal functioning. The maximum peak flow velocity in the left ventricular outflow tract measured 1.6 m/s (corresponding to maximal gradient of 10.3 mmHg), indicating no significant left ventricular outflow tract gradient. An increased peak flow velocity of 3.8 m/s (corresponding to a maximal gradient of 58 mmHg) together with the typical color Doppler pattern indicated severe stenosis of the descending aorta (Figures 1(a) and 1(b)). The parasternal short-axis views revealed a PDA (Figure 1(c)) and pulmonary to systemic flow ratio (Qp/Qs) was calculated as 1.8. Thorax computed tomography (CT) angiography showed that diameter of aorta at the preductal level is 20 mm, 11 mm at coarcted segment, and 23 mm at the postductal level. Also the diagnosis of PDA was confirmed with thorax CT angiography (Figure 2).

**Figure 3:**
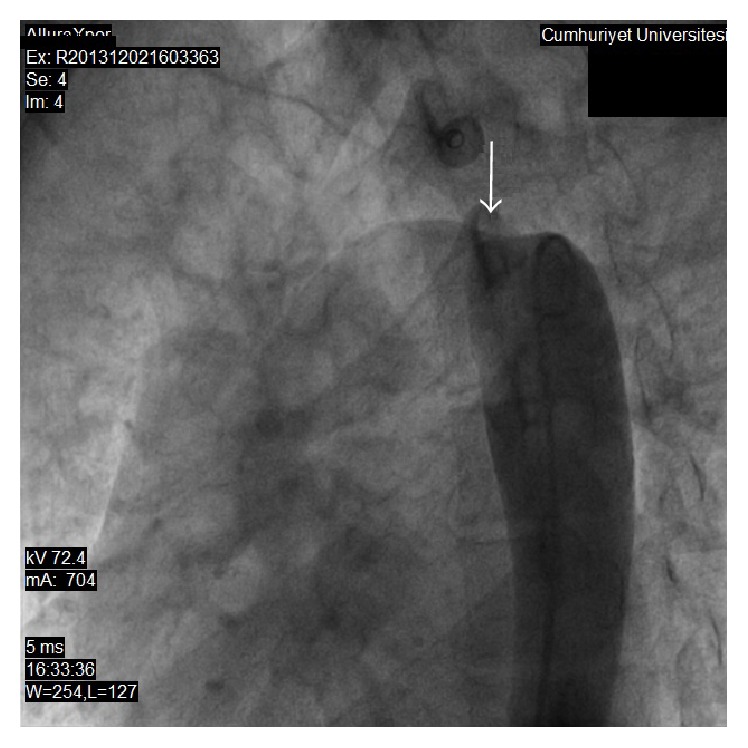
Aortography at postductal segment of descending aorta. Arrow indicates coarcted segment of aorta.

**Figure 4:**
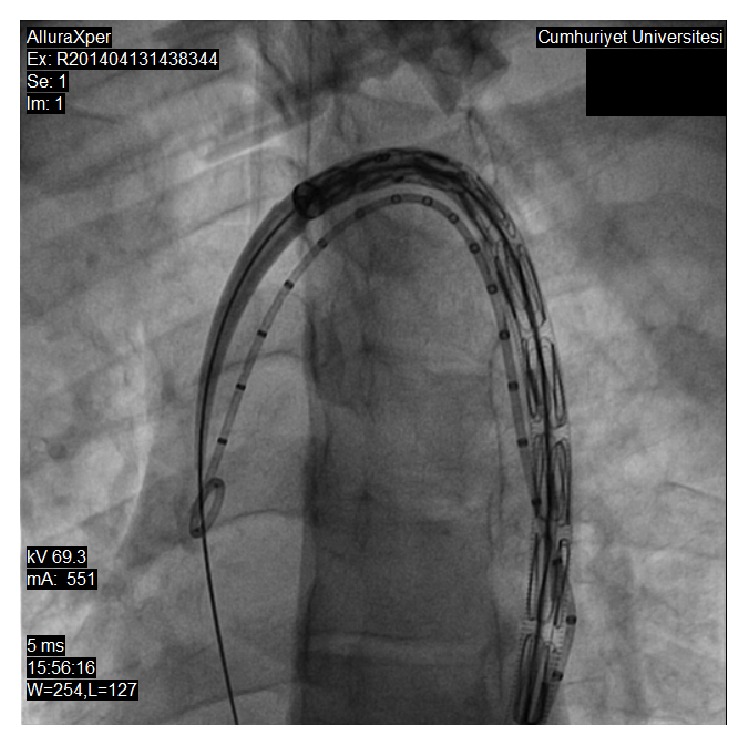
Thoracic stent graft was placed across the coarcted segment.

**Figure 5:**
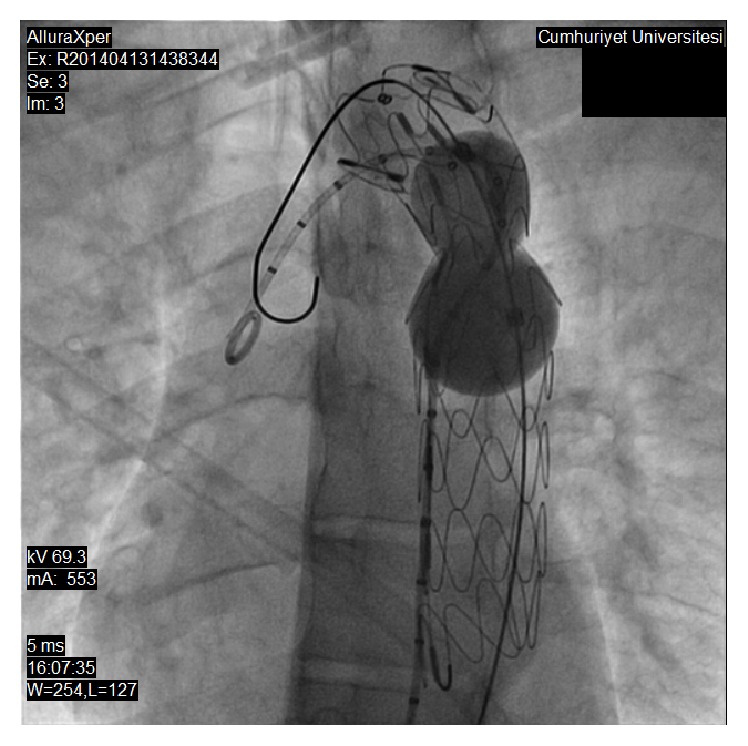
Postdilatation of thoracic stent graft.

**Figure 6:**
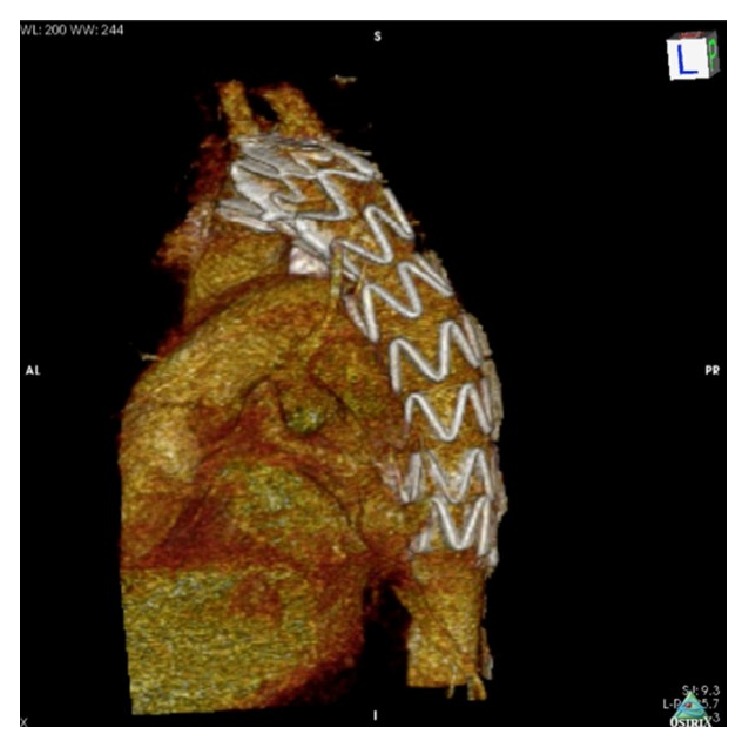
Thorax CT angiography showed good apposition of thoracic stent graft with complete exclusion of PDA.

Percutaneous treatment of the CoA and PDA with a generally preferred balloon expandable covered stent was planned. But balloon expandable covered stents dedicated for CoA were not available at those days. For this reason, we decided to use a self-expandable thoracic stent graft. Because of their small size detected on CT angiography, common femoral arteries (3.0 mm, both) and external iliac arteries (4.0 mm, both) were not suitable for thoracic stent graft implantation. So we decided to use left common iliac artery (CIA) via RP approach which was 5.5 mm in diameter (right CIA was 5.3 mm). The procedure was performed under general anesthesia. A 6-F pigtail catheter was placed via right femoral artery to the descending aorta, but the catheter could not pass the coarcted segment (Figure 3). The coarcted segment could be crossed using 0.035-inch hydrophilic guide wire (Radifocus, Terumo Corporation, Tokyo, Japan) and 5-F multipurpose diagnostic catheter (Cordis Corporation, Miami, Florida, USA). This catheter was changed with a 5-F marker pigtail catheter (Cook Inc., Bloomington, USA). Gradient across coarcted segment was measured as 85 mmHg. For retroperitoneal approach of left CIA, an incision was made in the skin of left lower quadrant of abdomen about 3 cm above from the inguinal ligament. Then, left CIA was reached and a 6-F introducer sheath was placed by Seldinger technique. Coarcted segment was passed with hydrophilic guidewire and 5-F multipurpose catheter via the sheath in left CIA. Hydrophilic guidewire was changed with extra stiff Amplatz guidewire (Amplatz Extra Stiff 0.035 inches, Boston Scientific, MA, USA). The sheath in left CIA was exchanged with a 22 Fr E-asy Plus hemostatic valved introducer sheath (Jotec GmbH, Hechingen, Germany). A 26 mm × 100 mm sized thoracic stent graft (Ankura, Lifetech Scientific Ltd., Shenzhen, China) was placed over the coarcted segment via the 22 Fr sheath (Figure 4). After confirmation of optimal position, the stent was released and then dilated with a balloon catheter (E-xpand, Jotec GmbH, Hechingen, Germany) with high atmosphere for optimal dilatation of the stenosed segment to exclude residual gradient and complete apposition of the stent (Figure 5). Control aortography confirmed that the coarcted segment is optimally dilated and the PDA is completely covered by stent graft without any leak. The measured gradient across coarcted segment after stent placement was 5 mmHg. Similar findings were confirmed by echocardiographic assessment. Also intravascular pressure difference between ascending aorta and femoral artery was 5 mmHg. The patient recovered well and was discharged three days after the procedure. Before discharge, control CT angiography showed fully expanded thoracic stent graft with complete exclusion of PDA (Figure 6). The patient was asymptomatic after two years.

## 3. Discussion

Simple coarctation in the absence of accompanying lesions (e.g., ventricular septal defect, mitral stenosis, and bicuspid aorta with stenosis) is the most common form in adults [7]. Complex coarctation with other defects (e.g., coarctation with PDA) is not common in young adults [10]. Our patient had complex coarctation with postductal PDA. Open surgical repair is still the gold standard therapy for totally occluded CoA, but due to significant collateralization through the intercostal arteries, these surgical patients increase the risk of bleeding complications. In addition, other early postoperative complications such as paradoxical hypertension, aortic dissection, left recurrent laryngeal nerve paralysis, phrenic nerve injury, and subclavian steal are still important [11]. Transcatheter closure of moderate-to-large ducts with occluder devices has been widely practiced in recent years [4]. Treatment of CoA associated with PDA depends on patient's age, features of coarcted segment, and the size of the duct [4]. Patients with small sized common femoral artery are not suitable candidates for endovascular interventions with big sized endovascular stent grafts. In these cases, CIA can be cannulated via RP or TP approaches [12]. The TP approach was historically the most practiced way. However, the RP iliac approach advocates the significant benefits of not entering the peritoneal cavity and avoiding a long middle incision [9, 12]. Consequently, the decreased incidence of paralytic ileus and pneumonia is expected. The reduced incidence of these complications may have led to a significant decrease in the length of the hospital stay. Many studies revealed that the RP approach is superior to the TP approach in many respects [9, 13].

Balloon expandable covered stents seem to be the ideal and simple percutaneous option for the cases with CoA plus PDA. Also these stents could be more suitable for this patient with small sized common femoral arteries, because the maximal sheath size for delivering these stents was 12 Fr. But these stents were not available in our hospital. Besides surgery which was refused by patient and her relatives, percutaneous PDA occlusion with duct occluder and balloon expandable bare stent dedicated for CoA or only balloon dilatation for CoA could be another option [4]. But our cardiovascular team (cardiologists and cardiovascular surgeons) decided to perform thoracic stent graft in the line of thought that similar result could be obtained as balloon expandable covered stent. On the other hand, disadvantages of thoracic stent graft as compared with balloon expandable covered stent are longer stent size and need for bigger vascular access. Although occlusion of the subclavian artery and the other arteries arising from aorta is a risk with long thoracic stent grafts, in case of rupture or dissection [14], further interventions may not be needed with these stents.

Middle aortic coarctation (MAC) is another rare type of CoA which is usually presented in youth or adolescence [15]. This type of coarctation comprises only 0.5–2.0% of all aortic coarctation cases [15]. MAC can cause renovascular hypertension like CoA [15], but it may be difficult to be diagnosed with transthoracic echocardiography, because of its location. Abdominal ultrasonography and abdominal CT angiography are usually the diagnostic tools for MAC. It should be suspected in patients with refractory hypertension, lower extremity claudication, and/or abdominal angina presentation [15]. Abdominal aorta was found to be normal on CT in our case.

This case is the first aortic coarctation plus patent ductus arteriosus case described in the literature which is treated by endovascular thoracic stent graft via retroperitoneal approach.

## 4. Conclusion

Thoracic stent graft can be used to treat CoA associated with PDA, but not alternative to balloon expandable covered stents. In case of need for bigger vascular access site than common femoral artery diameter, RP iliac approach is a good and safe option for endovascular interventions.
